# Somatic non-cancerous PIK3CA-related overgrowth syndrome treated with alpelisib in North America

**DOI:** 10.1007/s00109-020-02030-6

**Published:** 2021-01-03

**Authors:** Alexandre P. Garneau, Ludwig Haydock, Laurence E. Tremblay, Paul Isenring

**Affiliations:** Nephrology Research Group, L’Hôtel-Dieu de Québec du CHU de Québec, Department of Medicine, Faculty of Medicine, Laval University, 10 McMahon Street (Room 3852), Québec, QC, G1R 2J6 Canada

Overgrowth syndromes (OS) of the type presented by the Elephant Man (Joseph Merrick) in the late 1800’s are perceived as peculiar disorders. Yet, their causes are now well established, and their prevalence is probably underestimated. Virtually any tissues (fat, vessels, lymphatics, bone, viscera, etc.) can overgrow but to varying degrees among affected individuals [1].

Most forms of OS are caused by genetic/epigenetic defects during embryogenesis [1]. The proteins at play are typically involved in cell proliferation and have acquired an overactive state. They include members of the PIK3CA/AKT/mTOR^1^ signaling pathway as well as upstream or downstream effectors [1, 2]. A common form of OS is now referred to as PIK3CA-related overgrowth spectrum (PROS)^2^.

Due to the nature of the defects that are commonly at cause, i.e., gain-of-function mutations in specific regulatory intermediates, OS could be theoretically amenable to treatment through pharmacological inhibitors. Unfortunately, many of the compounds that would be of potential benefit in the management of such disorders can cause serious toxicity or are still under development.

In that regard, alpelisib (Novartis) stands as an exception by being a potent, selective, well-tolerated, and commercially available PIK3CA inhibitor for oral use [3]. During the last years, moreover, ~ 60 patients were treated for PROS with this drug under the care of Dr. Guillaume Canaud at *Necker*-*Enfants Malades* Hospital, and all have experienced regression over their overgrowths. Nineteen of them were also the object of a recent publication in *Nature* [2].

As it stands, there have been no scientific reports of patients to have received alpelisib for the management of PROS in North America. Recently, this drug was used by us for one such case, that of a 29-year-old Canadian woman, and led to a spectacular response. We are thus taking advantage of the current commentary section to share our experience with the medical community.

The defect was identified in a skin biopsy taken from an affected area. It consisted of a low allele frequency (4%) gain-of-function Y1025A mutation in PIK3CA. As for the clinical presentation (Fig. 1), it was characterized by (1) mesenteric lipomatous overgrowth with recurrent bouts of severe, opioid-dependent abdominal pain, (2) moderate hypertrophy of the left leg, and (3) a large port-wine stain over the right leg with a multitude of superficial and deep-seated venous malformations.

**Fig. 1:**
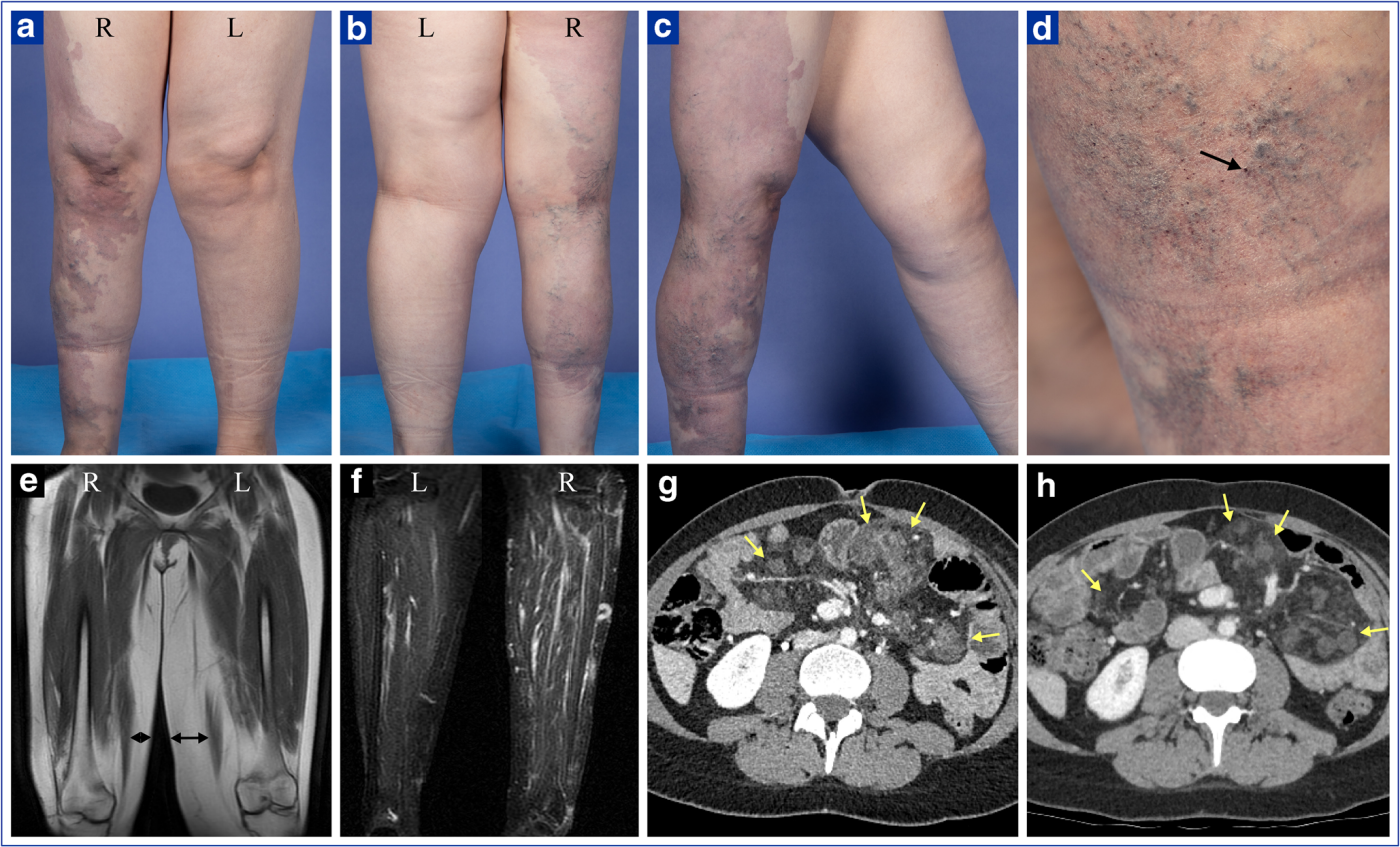
Clinical manifestations at baseline (**a** to **g**) and after 2 months on a PIK3CA inhibitor (H). (**a**–**d**) External appearance. Left leg is seen to be moderately enlarged and right leg to harbor a large nevus flammeus (port-wine stain) with multiple varicose veins and superficial hemangiomas (one of which is pointed by an arrow) below the knee. (**e**–**f**) Magnetic resonance imaging of lower limbs. Left leg is seen to exhibit subcutaneous overgrowth (**e**) and right leg multiple vascular malformations (**f**). (**g**, **h**) Enhanced CT scanning of abdomen. Mesenteric lipomatosis is seen on both panels (delineated by yellow arrows) but has reduced substantially in contrast enhancement and bulkiness 2 months after the PIK3CA inhibitor was begun (compare panel h to panel g). Note that the skin lesions had regressed only slightly after 2 months of treatment

Many of these manifestations had progressed steadily from early childhood to beginning of adulthood. Because of them, the patient had been unable to enter the labor market and had been poorly active whether it be socially or physically for several years. The venous malformations had also led to repeated episodes of pulmonary embolisms for which long-term anticoagulation was eventually required.

Four days after alpelisib was started at 150 mg/day^3^, abdominal pain had already begun to subside. Two months later, and at the cost of mild intermittent diarrhea, mesenteric overgrowth and use of analgesics had decreased by approximatively 50% (Fig. 1), while general well-being, quality of life, and socialization had improved drastically. In the next weeks, alpelisib will be increased progressively to a standard dose of 250 mg/day.

Pain is noticeably common in PROS and has also been found to improve under alpelisib therapy [2]. Given that it began to subside very rapidly once this therapy was initiated in our case, and at a time when tissue overgrowth could not have decreased substantially, one could postulate that PIK3CA overactivation causes tissue pain by driving nociceptive threshold downwardly or by exerting AKT/mTOR-dependent pro-inflammatory effects.

The prevalence (ρ) of PROS (ρ ~ 1:1,000,000) is probably grossly underestimated [1, 2] as other conditions have been shown to (or could) be PIK3CA-related in many cases. They include the hemihypertrophic syndrome of Klippel-Trenaunay (ρ ~ 1:100,000) and milder overgrowth disorders in the form of isolated digital hypertrophy, port-wine stains (ρ > 1:1000) and arteriovenous malformations^4^ [4, 5].

In our opinion, PIK3CA inhibition will eventually find relevance in other or more common conditions as a substitute to potentially invasive or debilitating procedures. Among others, such conditions could include PIK3CA-mutated facial capillary malformations or superficial skin cancers that could be managed safely if enzyme inhibition could be achieved topically [6].

In conclusion, this case of PROS should be seen as remarkable given that it is the first to have been treated in Canada with alpelisib and first one in North America to be the object of a scientific report for having received this drug. It is only a matter of time before OS is managed through precision medicine across all continents.

## Data Availability

Data will be made fully available upon request.

## Abbreviations

AKT: Protein kinase B
CLOVES: Congenital lipomatous overgrowth with vascular malformations, epidermal naevi and skeletal abnormalities
KRAS: Kirsten rat sarcoma viral oncogene
mTOR: Mammalian target of rapamycin
OS: Overgrowth syndrome
PIK3CA: Phosphatidylinositol 3-kinase type C alpha
RASA1: Ras GTPase activating protein
TEK: Tyrosine kinase with EGF homology domain

## Footnotes

1 PIK3CA is the p110α subunit of phosphatidylinositol 3-kinase in the PIKC3CA/AKT/mTOR transduction pathway. Upstream activators include KRAS and TEK/TIE2.

2 The prototypal form of PROS is known as CLOVES syndrome.

3 Alpelisib is the only PIK3CA inhibitor available commercially [3]. It was used off-label through the Managed Accessed Programs of Novartis after approval by Health Canada and started at a lower dose than usual to limit the possibility of intolerance.

4 These conditions have also been linked to signaling intermediates such as KRAS and RASA1 for which inhibitors are under development [4, 5].
